# Simultaneous growth of three-dimensional carbon nanotubes and ultrathin graphite networks on copper

**DOI:** 10.1038/s41598-019-48725-w

**Published:** 2019-08-28

**Authors:** Lee-Woon Jang, Jaeho Shim, Dong Ick Son, Hyunjin Cho, Luman Zhang, Jie Zhang, Mariela Menghini, Jean-Pierre Locquet, Jin Won Seo

**Affiliations:** 1 KU Leuven, Department of Materials Engineering, Leuven, B-3001 Belgium; 2 Korea Institute of Science and Technology, Institute of Advanced Composite Materials, Jeonbuk, 55324 Republic of Korea; 3 Chonbuk National University, Department of Organic Materials and Fiber Engineering, Jeonju, 54896 Republic of Korea; 4 KU Leuven, Department of Physics and Astronomy, Leuven, B-3001 Belgium

**Keywords:** Carbon nanotubes and fullerenes, Electronic properties and materials

## Abstract

A new way to simultaneously grow carbon nanotubes (CNTs) and ultrathin graphite on copper (Cu) foils has been investigated. This one-step growth process yields three-dimensional networks of CNTs on graphitic layers (3D CNTs/G) on Cu foils. Their synthesis conditions and growth mechanism are discussed in detail taking their structural properties into account. Individual CNTs and the 3D CNTs/G networks by means of an *in-situ* conductive atomic force microscope inside a scanning electron microscope are electrically characterized. Time-resolved photoluminescence demonstrated fast charge transfer and high carrier collection efficiency superior to two-dimensional ultrathin graphite only. Their facile and tunable growth and excellent electrical properties show that the 3D CNTs/G are strongly attractive for various applications such as solar cells, sensors, supercapacitors, photovoltaics, power generation, and optoelectronics.

## Introduction

Carbon based nanostructures such as carbon based quantum dots, carbon nanotubes (CNTs), graphene oxide, graphite and graphene have a wide structural variety in the range from zero-dimension to three-dimension (3D)^1–4^. In particular, 3D carbon nanostructures including stacking of CNTs or graphene oxide, composites of CNTs and graphene enable volumetric modifications combined with conductivity and flexibility^5–8^. Especially, hybridization of 3D carbon nanostructures and other active materials has demonstrated its great advantage in solar cell, capacitor, and energy storage applications^9–11^. CNTs in perovskite absorbers, for instance, presented excellent photo-generated charge transport^9^. CNTs and graphene mixtures with electrolytes increased volumetric energy density and resulted in improved supercapacitance performance^10^. Lithium-sulfur with CNTs and graphene mixtures also enabled high-rate, thermally and chemically stable flexible batteries^11^. In these hybrid applications, efficient charge transfer, high volumetric area, and interspaces to incorporate active materials and to allow ion exchanges are key factors to maximize charge collection efficiency.

For 3D CNTs and graphene hybrid mixtures, common fabrication methods have been mixing, stacking and coating of CNTs or graphene together with active materials^12^. Though it is a simple and effective technique, their distribution, fraction, and thickness are difficult to control. Recently, alternative growth methods of 3D CNTs on graphene were reported. Aligned CNTs were grown on the underlying graphene layer, and their volumetric area was controlled by the height of CNTs^13,14^. Seamless structure without defect-traps between CNTs and graphene provided high carrier transportation^15^. In addition, high mobility and fast decay behaviour of carriers in 3D carbon structures enabled performance improvements in solar cells, sensors, catalysts, capacitors, and energy storages^12–19^.

However, the growth of the aligned 3D CNTs on graphene is still challenging due to the difficulty in tuning the precise growth conditions for both CNTs and graphene, and in controlling the structural properties including the length and the density of CNTs^13–18^. It requires numerous process steps involving sequential growth of graphene and CNTs, surface treatments for uniformly distributed catalysts and/or lithographic process on graphene or CNTs. These multiple and complicated procedures rather hamper fast and reliable production of the 3D CNT on graphene and, consequently, limit the implementation of the latter in devices.

In this work, we report the one-step growth of the 3D CNT on graphitic layers on Cu foils. Without additional lithography and/or catalyst deposition, vertical CNTs and multilayer graphene were simultaneously grown on Cu foils. These graphitic layers are considered as ultrathin graphite due to multiple stacked (about 20) layers of graphene^20^. The growth mechanism and the controllability of the CNT length and density were investigated. Structural characterizations of the 3D CNTs on ultrathin graphite (3D CNTs/G) networks were carried out by Raman spectroscopy and transmission electron microscopy (TEM). Electrical properties between CNTs and ultrathin graphite were determined by using a conductive atomic force microscope (AFM) integrated in a scanning electron microscope (SEM). Charge transfer ability of the 3D CNTs/G was confirmed by time resolved photoluminescence (TRPL) and compared with ultrathin graphite only. Our results demonstrate that the 3D CNTs/G networks grown by the one-step growth represent an excellent carrier transporting material due to the increased volumetric area and efficient carrier collection through the CNTs. Together with the advantage of the simple growth process, the 3D CNTs/G are highly promising for future carbon-based catalyst, photovoltaic, sensor, electronic and energy storage devices.

## Results and Discussion

Compared to the growth of graphene, CNTs require different growth parameters including a lower reactor temperature and metal nanoparticles as catalysts. Especially, the latter plays the most important role for the CNT growth because the nanoparticles enable the catalytic reaction, the confined seeding and finally the formation of CNTs^21^. For efficient seeding of CNTs, nanoparticles of 3d transition metals such as iron, nickel, Cu, and their alloys with high carbon adsorption rate are used^22,23^. Consequently, so far the 3D CNTs on graphene networks have mostly been obtained in two separate CVD processes for graphene and CNTs, respectively.

**Figure 1:**
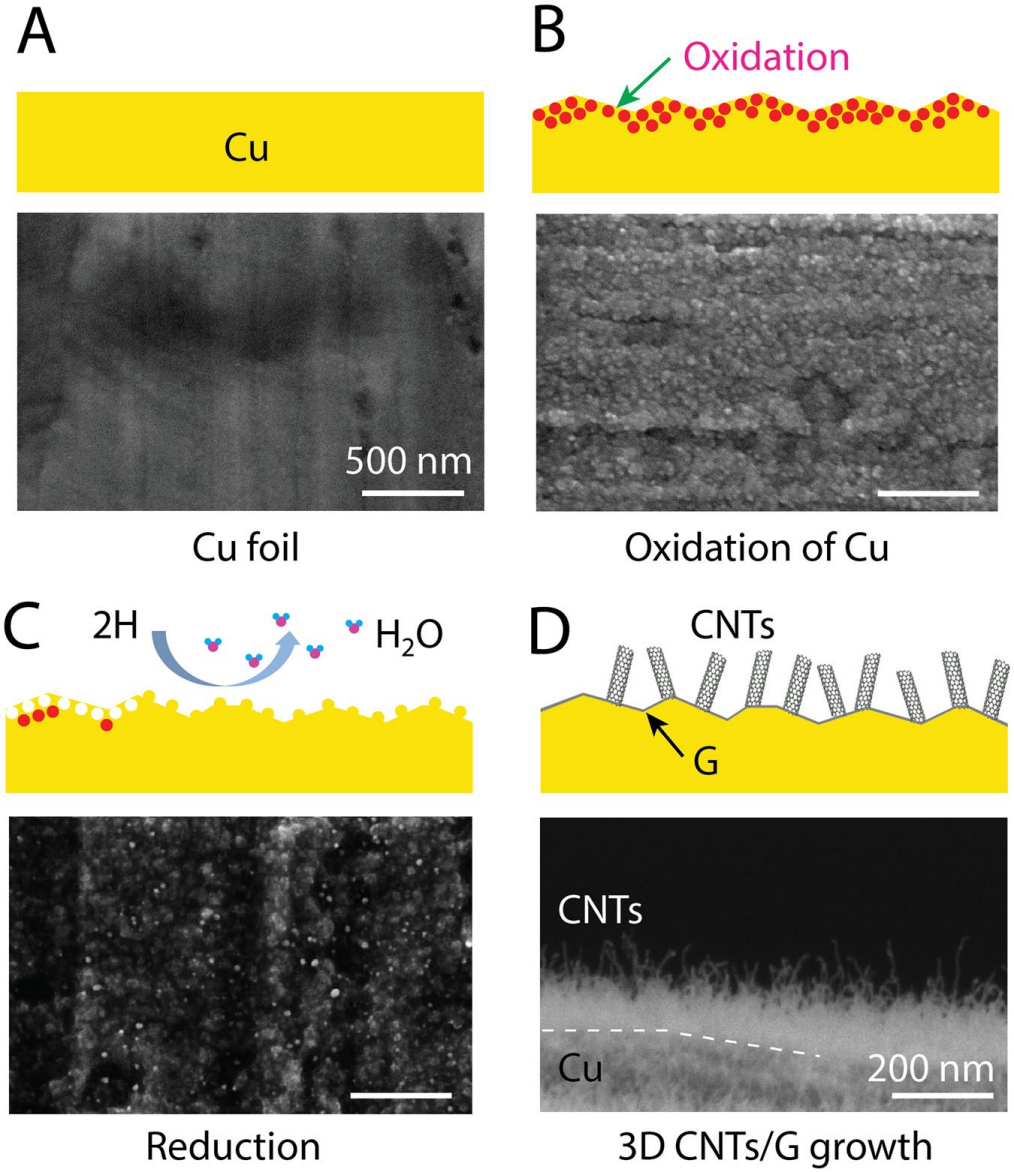
Schematic drawing illustrating the growth procedure of the 3D CNTs/G networks and respective SEM images demonstrating the change of the Cu surface after each step. (**A**) Pristine Cu foil, (**B**) oxidized Cu surface after annealing on a hot-plate, (C) surface after the reduction step in H_2_-rich atmosphere, (**D**) grown 3D CNTs/G networks.

However, we demonstrate that the CNT growth can be achieved directly from oxidized Cu foils together with graphene growth in the same process. Figure 1 summarizes the growth procedure. At first, a Cu foil was annealed on a hot-plate at 190 °C for 10 min (Fig. 1B). The Cu surface oxidized during this heating step and roughened due to the transition from Cu to oxide phases^24^. As confirmed by X-ray diffraction (see Fig. S2), various phases such as CuO, Cu_2_O, and Cu_4_O_3_ were formed. The oxidized Cu foil was loaded in a horizontal CVD reactor for the growth. During the temperature ramping and annealing steps, the oxide layer was reduced due to the exposure to hydrogen and turned back into the metal Cu surface. This reduction process by hydrogen left behind a roughened surface with nanoparticles, as illustrated in Fig. 1C. According to previous reports, these nanoparticles formed during the reduction process can also promote the seeding of graphene^25–27^. With continuous supply of acetylene and CO_2_, the edges of the surface or nanoparticles become saturated with carbon atoms and, finally, CNTs emerge^28^. At the same time, carbon atoms are also absorbed at the relatively flat Cu surface leading to formation of graphitic layers. A SEM image of a free-standing 3D CNTs/G confirms the presence of CNTs on ultrathin graphite (Fig. 1D). We identified the oxidation and the reduction procedure of Cu foils prior to the growth as the most important key factor for this one-step growth of the 3D CNTs/G. In contrast, pristine Cu foils without the initial oxidation step yielded only ultrathin graphite, and CNTs could not be obtained.

**Figure 2:**
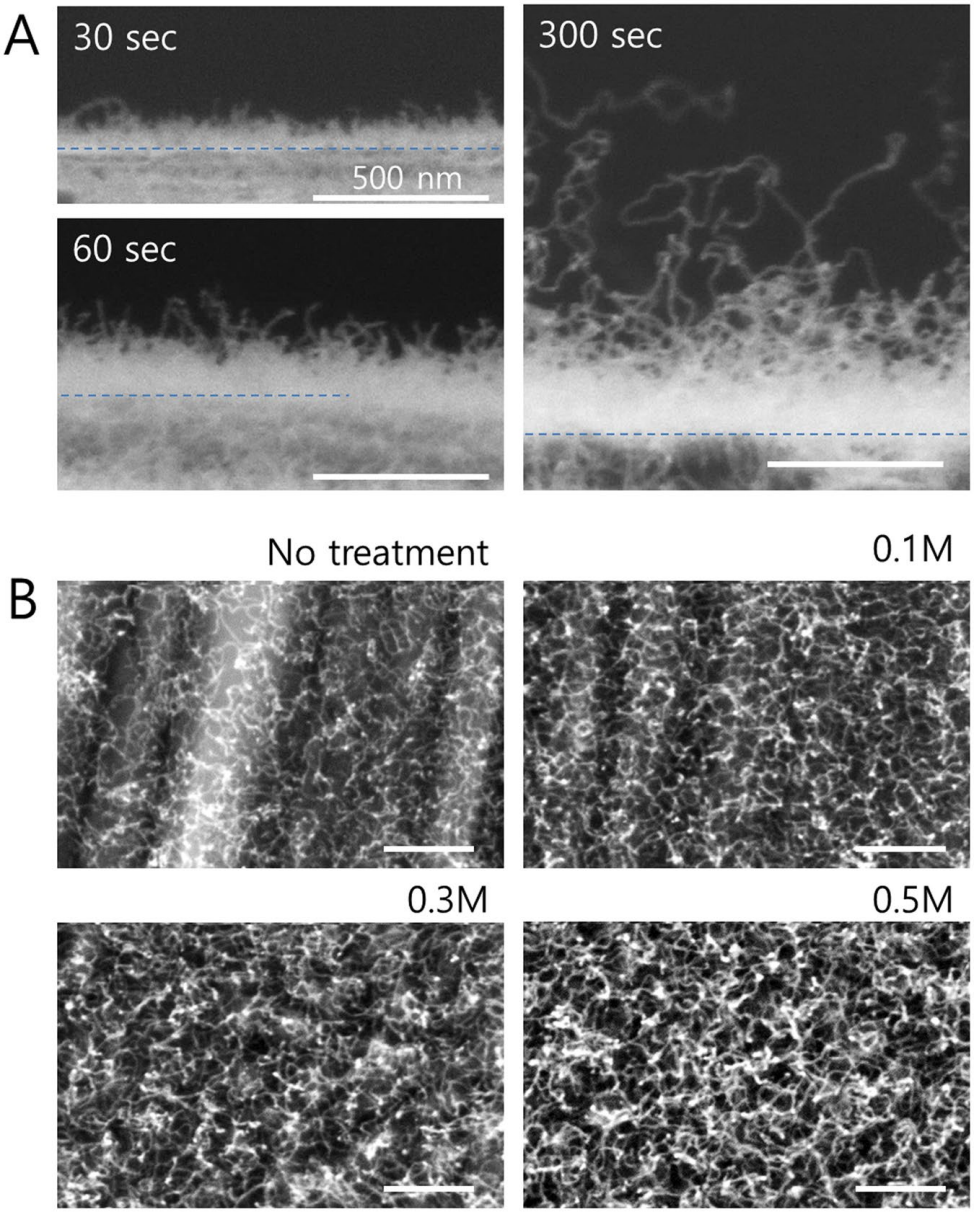
SEM images demonstrating the variation of the length and density of CNTs. (**A**) Cross-sectional images showing the different CNT length as a function of the growth time, (**B**) top view illustrating the CNTs density as a function of the ammonium persulfate concentrations from 0 M to 0.5 M.

For a more detailed understanding of the growth, we also investigated the dependence of the CNT length and the density of CNTs. As shown in Fig. 2A, the length of CNTs increases with the growth time, and the growth rate can be estimated to approximately 250 nm/min. Although CNTs are not vertically aligned but highly entangled, they are densely grown from the Cu surface up to a height of 300 nm. Longer CNTs sparsely stick out of the dense mat reaching an additional length up to 1000 nm. As catalyst particles could not be detected at the tip of CNTs by SEM and by TEM, we assume a root-growth type mechanism for CNTs growth^28^. Fig. 2B shows the top view of the Cu foils after the 3D CNTs/G growth as a function of the concentration of ammonium persulfate used for cleaning of Cu foils. With increasing concentration of ammonium persulfate from 0.1 M to 0.5 M, the surface roughness of Cu foils gradually increased (see Fig. S4). Particularly after oxidation and reduction processes, the density of small nanoparticles increased leading to a higher density of CNTs as demonstrated in Fig. 2B.

**Figure 3:**
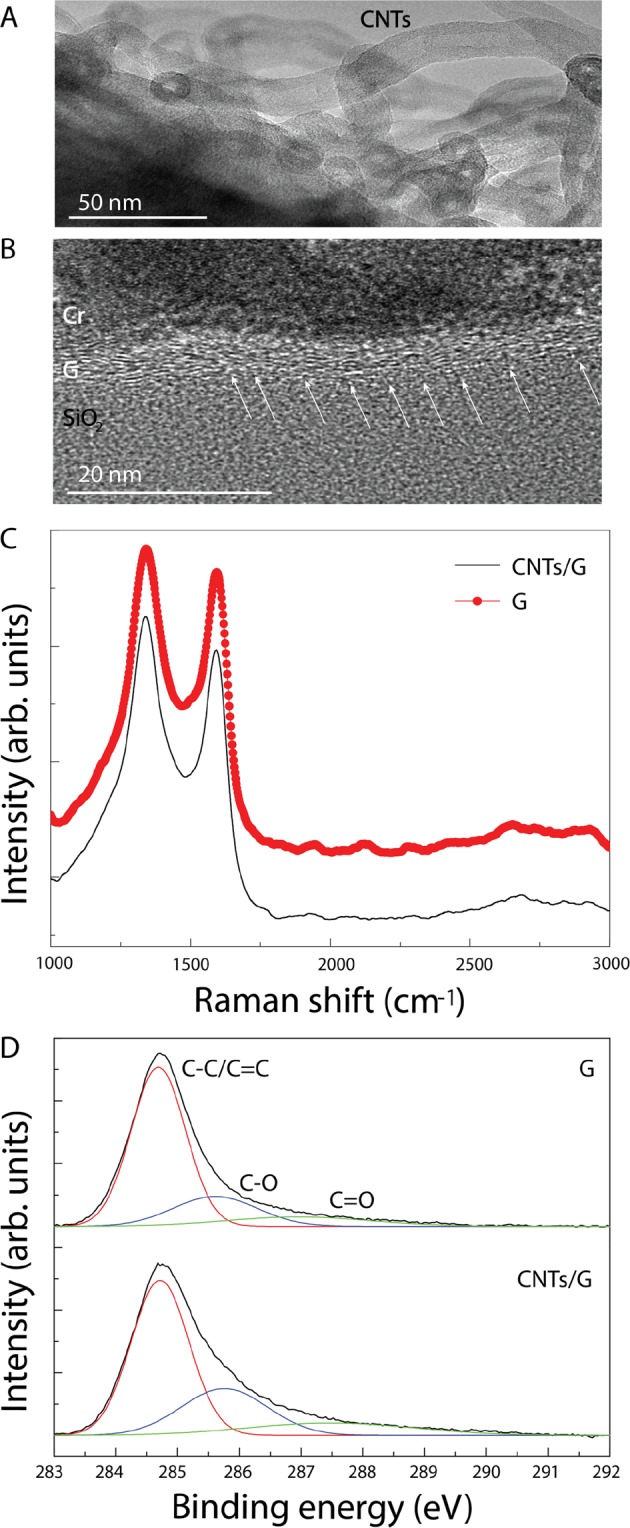
Structural and chemical characterizations of ultrathin graphite and the 3D CNTs/G networks: (**A**) TEM image of the CNTs, (**B**) TEM image of the ultrathin graphite layer from the 3D CNTs/G networks, (**C**) Raman spectra of the 3D CNTs/G and ultrathin graphite, (**D**) XPS spectra of the 3D CNTs/G and ultrathin graphite showing the C–C/C=C, C–O, and C=O bonds.

Structural properties of the 3D CNTs/G are determined by TEM and Raman spectroscopy. Extensive analysis of a series of TEM images of the CNTs revealed that the diameter of individual CNTs is around 25 nm and the wall thickness is 7~8 nm. In Fig. 3A, the top of the hollow inner core of CNTs can be seen as well as the inner structure containing a bamboo-like structure, which explains the highly entangled structure of the CNTs^29^. As can be seen in the cross-sectional TEM image (Fig. 3B), the 3D CNTs/G networks contain a multiple layered structure with a thickness of approximately 8~10 nm (Fig. 3B). This thickness of carbon structures corresponds to approximately 20 layers of graphene, and it is normally considered as ultrathin graphite^20^. Especially after etching the Cu substrate away, the typical continuous shape of ultrathin graphite in the free-standing 3D CNTs/G networks becomes apparent (see Fig. S3). The high defect density in ultrathin graphite of the 3D CNTs/G is obvious considering that the 3D CNTs/G was grown at 750 °C, which is significantly lower than the optimum growth temperature for high quality graphene^23,30^. Moreover, it was reported that the presence of CO_2_ in the oxidative dehydrogenation reaction promotes incorporation of structural disorders yielding defect densities up to 20%^31,32^. In Fig. 3B several structural discontinuities in the graphitic structures are marked by white arrows. The roughened surface of Cu also seems to promote structural defects in the ultrathin graphite layer. Nevertheless, the graphitic layer is continuous covering the entire Cu surface. Figure 3C shows the representative Raman spectra obtained from the 3D CNTs/G and additionally from an ultrathin graphite sample without CNTs. Both spectra are denoted by “CNTs/G” and “G”, respectively. The latter was grown, for comparison, at the same temperature but without oxidation of the Cu substrate in order to suppress the CNTs growth. In the Raman spectra, two dominant features appeared at around 1340 cm^−1^ and 1583 cm^−1^, which correspond to the D band for structural disorders and the G band referring to E_2_g mode, respectively. The low growth temperature of 750 °C and the oxidative dehydrogenation reaction of CO_2_ and C_2_H_2_ do not represent ideal condition for the synthesis of high quality graphene. Therefore, the Raman spectra look very similar to conventional CNTs or graphene oxide, showing a high D peak intensity^32,33^. Calculated D/G ratios of ultrathin graphite and CNTs/G correspond to 1.08 and 1.13, respectively. It seems that the presence of entangled CNTs leads to a higher density of structural defects. This observation is supported by the XPS spectra shown in Fig. 3D, which exhibit a relatively low intensity for the peaks correlated with the C–O and C=O bonds in ultrathin graphite. Calculated percentages of C–O and C=O bonds in ultrathin graphite are about 20% and 10.9% respectively, while the 3D CNTs/G shows 26.7% of C–O and 13.6% of C=O. Comparable results have been observed in functionalized CNTs and reduced graphene oxide layers^32–34^.

Intuitively, higher growth temperatures may lead to better structural properties of the carbon structures. However, the growth at high temperatures significantly increases the difficulty of controlling the CNT length/density, although the defect density didn’t change as observed in the Raman spectra shown in Fig. S4. In order to minimize defects while maximizing the quality of 3D CNTs/G, alternative route such as using methane as a carbon source is more likely to be efficient.

**Figure 4:**
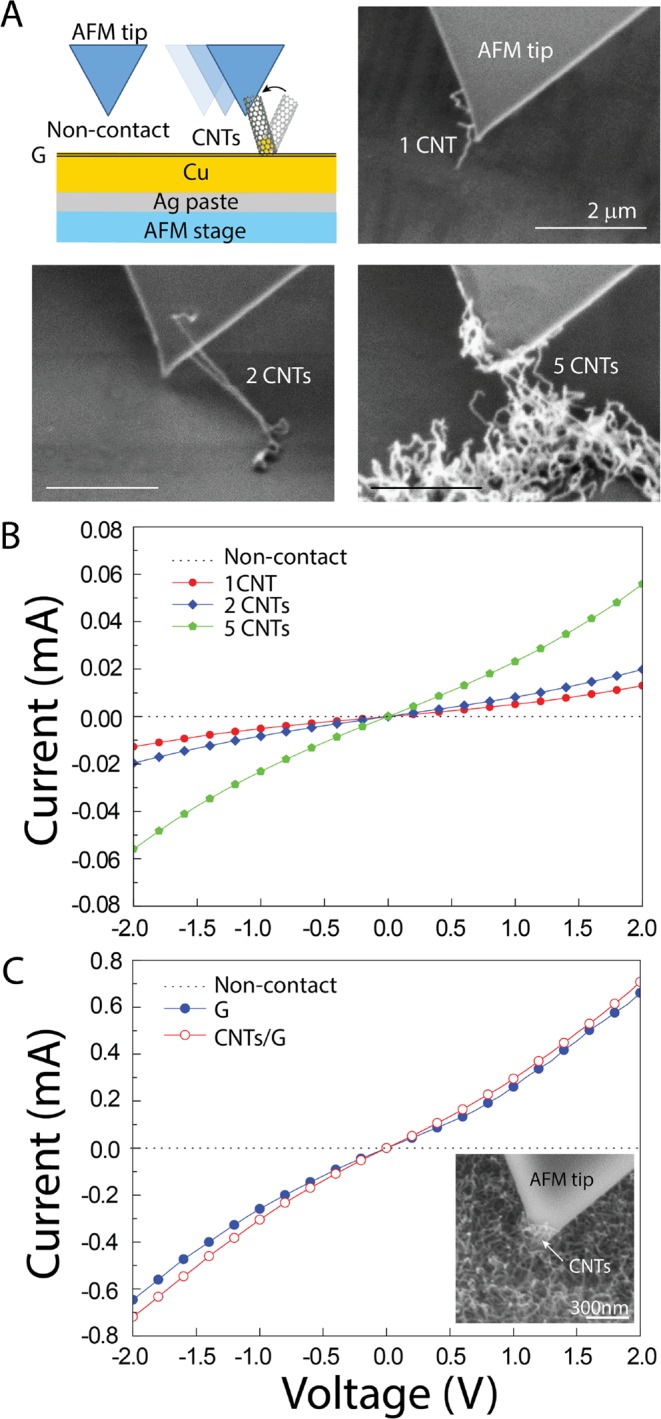
Electrical properties measured by means of the combined AFM/SEM system. (**A**) Schematic drawing illustrating the approach to individual CNTs in non-contact mode, and SEM images of the AFM tip with contacted CNTs. (**B**) I–V curves measured from 1, 2 and 5 individual CNTs, (**C**) I–V curves obtained from ultrathin graphite and from the 3D CNTs/G network in contact with the tip. The inset shows a SEM image of the 3D CNTs/G in direct contact with the AFM tip. The non-linear behaviors of the I–V curves are caused by an electrostatic contact of the AFM tip without metal bonding.

Electrical properties of individual CNT and ultrathin graphite have been measured by using a conductive AFM integrated inside a SEM. This hybrid AFM-SEM system allows operating the AFM in the range of nanoscale while monitoring the AFM tip and the sample in real-time. With this system, the attachment of CNTs on the AFM tip can be observed while directly measuring I–V curves. Therefore, it is possible to measure individual CNTs as well as CNT bundles. The I–V curves were measured with voltages in the range from −2 V to 2 V. For an open-circuit system (non-contact), the distance between the Cu surface and the AFM tip was approximately 200 nm. When the AFM tip moved close to the CNT, the CNT was attracted to the tip due to the electrostatic interaction and made direct contact, as highlighted in Fig. 4A. The average current value of a single CNT/G was found to be approximately 11 μA at 2 V. With increasing number of CNTs on the AFM tip (Fig. 4B), the measured current increased. One advantage of the 3D CNTs/G is that several individual, long and flexible CNTs stick out (see Fig. 2A) and can easily touch the AFM tip and make direct contact for the measurement. Figure 4C shows I–V curves obtained from ultrathin graphite and the 3D CNT/G samples. Ultrathin graphite and the 3D CNTs/G networks yielded a current of 0.62 mA and 0.71 mA at 2 V, respectively. In the latter case, the current increased by approximately 15% because the presence of high density CNTs increased the contact area to the AFM tip. Figure 4C inset shows the SEM image of the AFM tip in contact with the 3D CNTs/G network.

**Figure 5:**
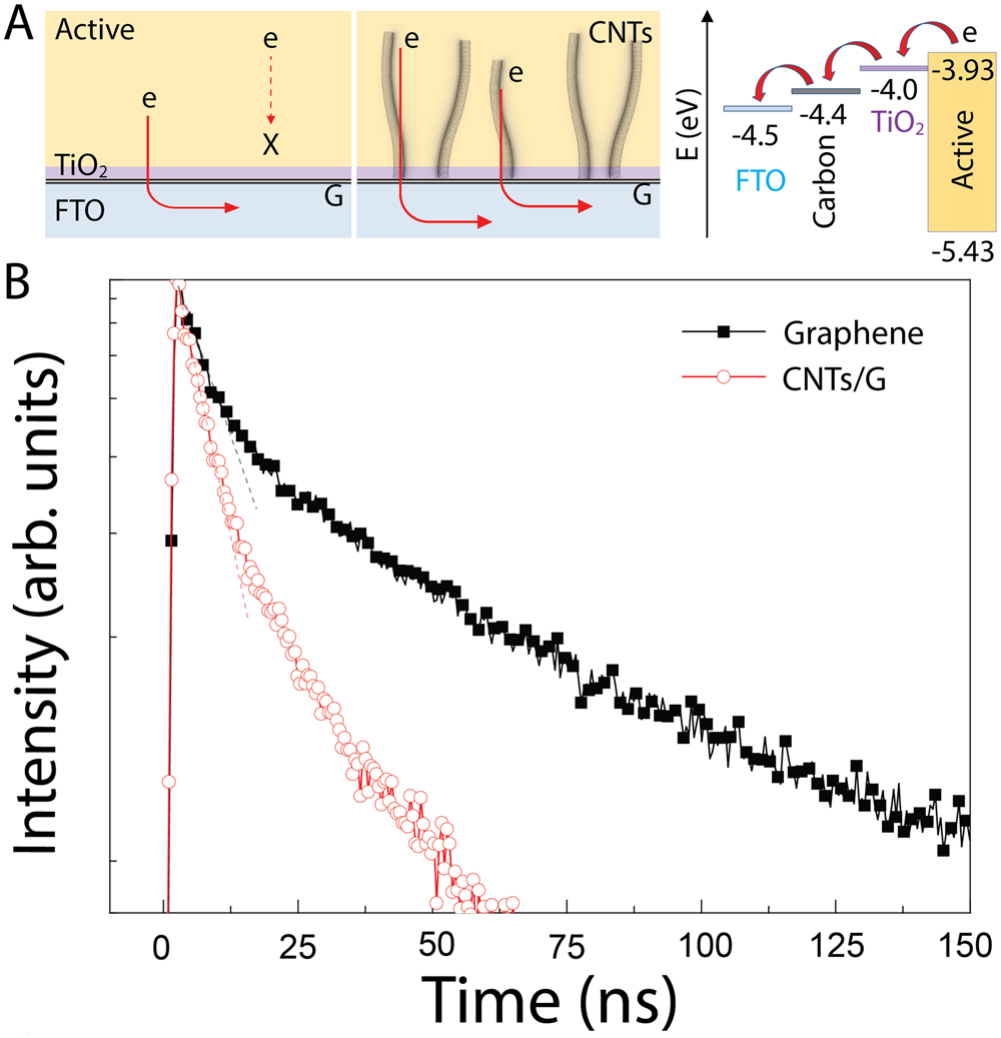
Performance of graphene and the 3D CNTs/G based perovskite device structures. (**A**) Schematic drawing of the devices highlighting the carrier transportation in both devices, and the energy band diagram for the 3D CNTs/G based perovskite device. (**B**) Carrier decay profiles of graphene and the 3D CNTs/G based devices obtained from TRPL measurements.

Obviously, the conductive 3D CNTs/G has the advantage of higher surface area and efficient carrier collection in optoelectronic devices^9,12,19^. As illustrated in Fig. 5A, the vertical CNTs, which are embedded inside the active layer, represent an efficient pathway for the carriers. Based on this concept, we investigated the carrier transport in the 3D CNTs/G with an active layer of CH_3_NH_3_PbI_3_. For comparison, also an ultrathin graphite sample with the same active layer was studied. A conventional perovskite optoelectronic device consists of an active layer of CH_3_NH_3_PbI_3_, electron and hole transport layers^9,35^. The energy band diagram was drawn on the basis of the work function: carbon nanostructures (−4.4~−4.2 eV), TiO_2_ (−4.0 eV), and perovskite (−3.93 eV)^36,37^. Due to a gradual lowering of the energy level of the carbon nanostructures and TiO_2_ compared to the active layer, the photo-generated carriers in the active layer easily relax through the vertical CNTs and can be transferred to the FTO electrode^12,19^. The lifetime of photo-generated carriers was analyzed from TRPL measurements as shown in Fig. 5B. The estimated lifetime of the carriers in ultrathin graphite and the 3D CNTs/G based structures is approximately 17 ns and 10 ns, respectively. The fast carrier movement by the 3D CNTs/G can generally be explained by two factors, which are the defect density and the carrier collection^35,38^. The Raman measurements confirmed that the 3D CNTs/G has the high defect density of 4.4% which is significantly higher than that in ultrathin graphite. Moreover, the XPS results indicated that the presence of C–O groups is 10.6% higher in the 3D CNTs/G. This increased defect density in the 3D CNTs/G can increase a nonradiative recombination rate of the photo-generated carrier and lead to a shorter lifetime^38^. In addition, the conductive CNTs of the 3D CNTs/G enabled the carrier collection vertically and also provide high surface area. The vertical collection behavior can reduce carrier trapping and increase the carrier lifetime in the active layer^35^. Both high nonradiative recombination and carrier collection of the 3D CNTs/G are the major reasons of the fast carrier movement, and consequently improved their performance compared to the only- ultrathin graphite based structure as shown in Fig. S5^9,13,14,19^.

For a deeper understanding between the active layer and the 3D CNTs/G structures, systematic investigations are required in order to analyze the device performance including incident photon-to-current behavior, impedance property, and a quantum yield characteristic. Nevertheless, our results clearly show that the conductive 3D CNTs/G networks directly promote the carrier collection by overcoming the drawbacks such as transmittance, surface area, and carrier diffusion issues. We believe that the 3D CNTs/G networks obtained in our single-step procedure are highly promising for further advancement of the 3D carbon based devices in optoelectronics, photovoltaics, sensors, and batteries.

## Methods

The 3D CNTs/G was produced on Cu foils by chemical vapor deposition. The Cu foils purchased from Alfa Aesar (0.025 mm thick) were at first cleaned in 0.1 M ammonium persulfate for 30 sec and washed in deionized water. Cleaned Cu foils were annealed on a hot-plate at 190 °C for 10 min to oxidize the Cu surface. The oxidized Cu foil was loaded into the horizontal reactor (Carbolite-Gero), with a quartz tube diameter of 4 cm and a uniform heating region of 30 cm, and annealed at 750 °C under atmospheric pressure in a flow of Ar and H_2_ with a flow rate of 500 and 100 sccm, respectively. After annealing for 5 min, both acetylene and carbon dioxide were introduced into the reactor simultaneously with a flow rate of 15 sccm while keeping the same Ar and H_2_ flows. After the growth of the 3D CNTs/G, the sample was cooled with a cooling rate of 7 °C/min under the continuous Ar flow of 500 sccm.

Structural morphologies of the 3D CNTs/G were characterized by SEM (Philips XL30 FEG) operating at 10 kV. For the preparation of the cross-sectional TEM samples, a protective chromium (Cr) layer and epoxy were deposited on the 3D CNTs/G, and focused ion beam (FIB, FEI Nova Nanolab 600) operating at 5~30 kV with Ga ions was used to prepare thin sliced TEM samples. TEM was performed to visualize the inner structure of ultrathin graphite and CNTs by means of a Tecnai G2 F20 (FEI) and an ARM200F (JEOL), both operating at 200 kV. For Raman spectroscopy, Cu foils under the 3D CNTs/G were etched in 0.3 M ammonium persulfate. The remaining 3D CNTs/G was washed in deionized water and then transferred onto SiO_2_/Si substrates. Raman spectra were obtained with a green laser of 532 nm wavelength and 0.2 mW power (Senterra, Bruker Optics). The crystal structure of Cu and oxide layers were identified by using a Philip X’Pert X-ray diffractometer operating at 40 mA and 40 kV, using a monochromatic Cu K_α1_ radiation. X-ray photoelectron spectroscopy (XPS) was performed in a SPECS XPS system with a monochromatic Al source (E = 1486.7 eV) and energy resolution of 2 meV. The *in-situ* measurement system including the conductive AFM (Semilab) inside a SEM (Carl Zeiss Merlin) was used to measure electrical properties of CNTs and graphene. All samples were mounted on the conductive stub holder with a silver paste, and the conductive AFM tip (model: SCM-PIT-V2, tip radius: 25 nm) was directly contacted to the sample using the DC mode. The current-voltage (I–V) curves were characterized using a Keithley 4200 parametric analyzer.

FTO glass with a sheet resistance of 8 ohm/cm^2^ (purchased from Solaronix) was cleaned by ethanol and deionized water. The synthesized 3D CNTs/G as well as the only-ultrathin graphite samples were separately transferred on the FTO glass after removing the Cu foil by treating with 0.3 M ammonium persulfate. For improving the wettability of organic perovskite active layer, a 50 nm thick mesoporous TiO_2_ layer was deposited on the transferred ultrathin graphite and the 3D CNTs/G on FTO glass substrates by spin coating and baked at 150 °C for 30 min. CH_3_NH_3_I and PbI_2_ (Aldrich) were dissolved in a mixture solution of dimethylformamide and dimethyl sulfoxide with a volume ratio of 3:1. This organic active layer with a thickness of 700 nm was spin-coated and then annealed at 100 °C for 10 min. TRPL was performed under pulse excitation of ultrathin graphite and the 3D CNTs/G based devices (pulse width of ~5 ps, center wavelength of 500 nm). All decay lifetimes were calculated by using the supplied software of Fluorescence Division.

## Supplementary information

Supplementary info
