# Supplementary material for: Simultaneous growth of three-dimensional carbon nanotubes and ultrathin graphite networks on copper
Source: Sci Rep. 2019 Aug 28;9:12344. doi: 10.1038/s41598-019-48725-w (PMC6713782; doi:10.1038/s41598-019-48725-w)
Supplement: Supplementary file 1 — Supplementary info [file 41598_2019_48725_MOESM1_ESM.doc]

**Simultaneous growth of three-dimensional carbon nanotubes and ultrathin graphite networks on copper**

Lee-Woon Jang^1^, Jaeho Shim^2^, Dong Ick Son^2^, Hyunjin Cho^3^, Luman Zhang^1^, Jie Zhang^1^, Mariela Menghini^4^, Jean-Pierre Locquet ^4^ and Jin Won Seo^1^*

^1^ KU Leuven, Department of Materials Engineering, Leuven, B-3001, Belgium

^2^ Korea Institute of Science and Technology, Institute of Advanced Composite Materials, 55324, Republic of Korea

^3^ Chonbuk National University, Department of Organic Materials and Fiber Engineering, 54896, Republic of Korea

^4^ KU Leuven, Department of Physics and Astronomy, Leuven, B-3001, Belgium

*[maria.seo@kuleuven.be](mailto:maria.seo@kuleuven.be)

**Supporting materials**

Figure S1. X-ray diffraction results of the pristine Cu and oxidized Cu foils. The oxide phases are indicated.


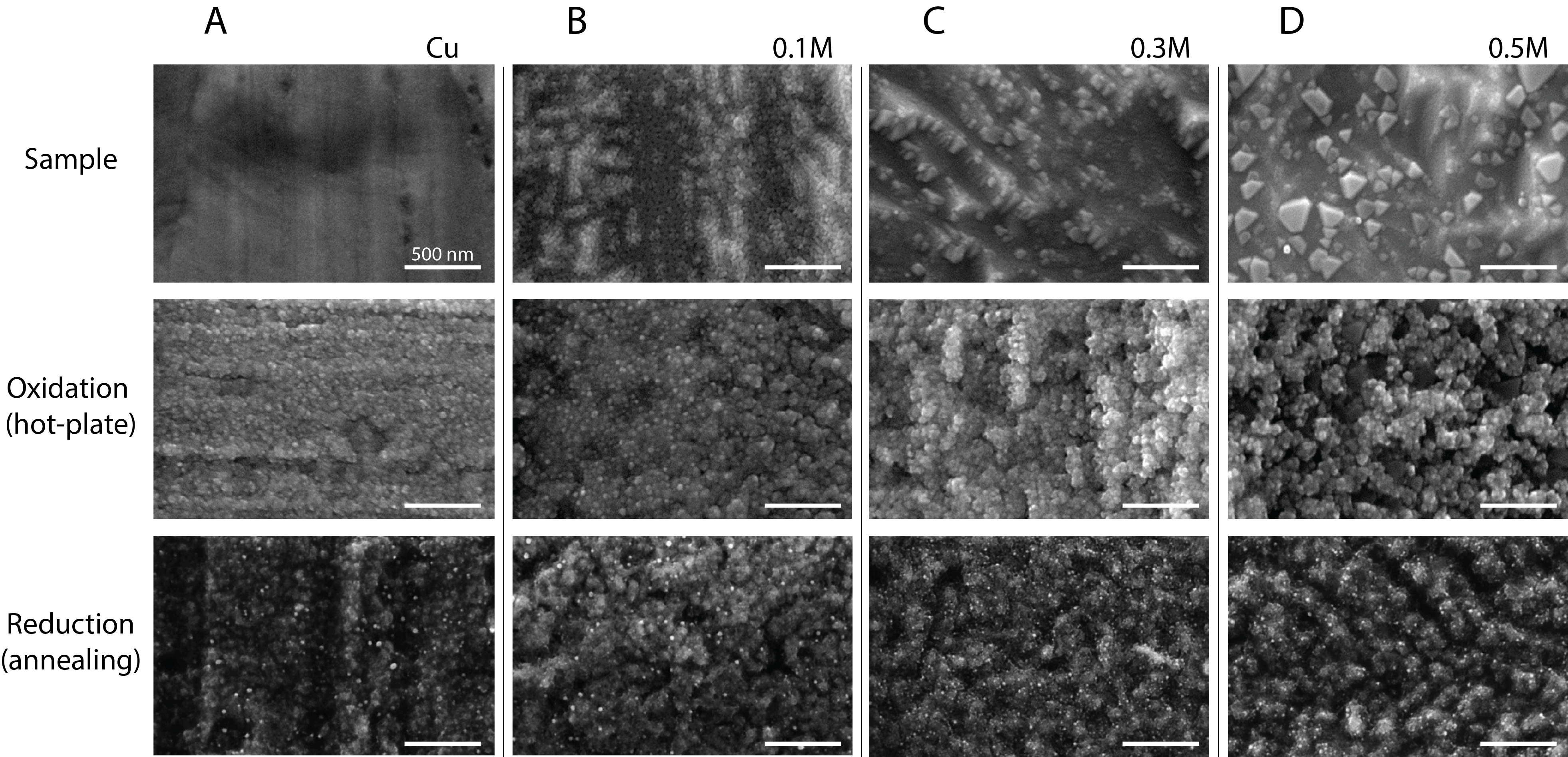


Figure S2. SEM images of the Cu surface morphologies after chemical etching, oxidation and reduction. (A) Surface changes of the pristine Cu foil, (B) 0.1M ammonium persulfate treated Cu foil, (C) 0.3M ammonium persulfate treated Cu foil, (D) 0.5M ammonium persulfate treated Cu foil. All Cu foils are oxidized on a hot-plate at 190 ^o^C for 10 min, and then reduced by H_2_ during the annealing step in a quartz tube.


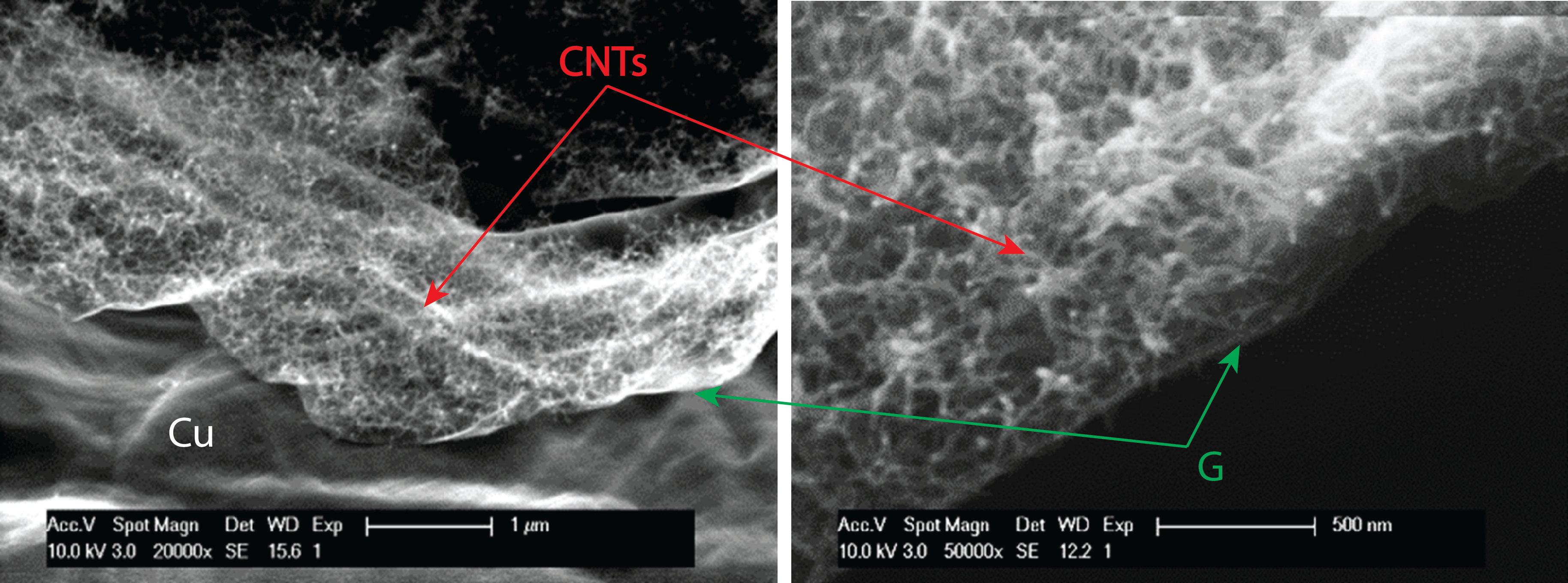


Figure S3. SEM images of the free-standing CNTs/G networks.


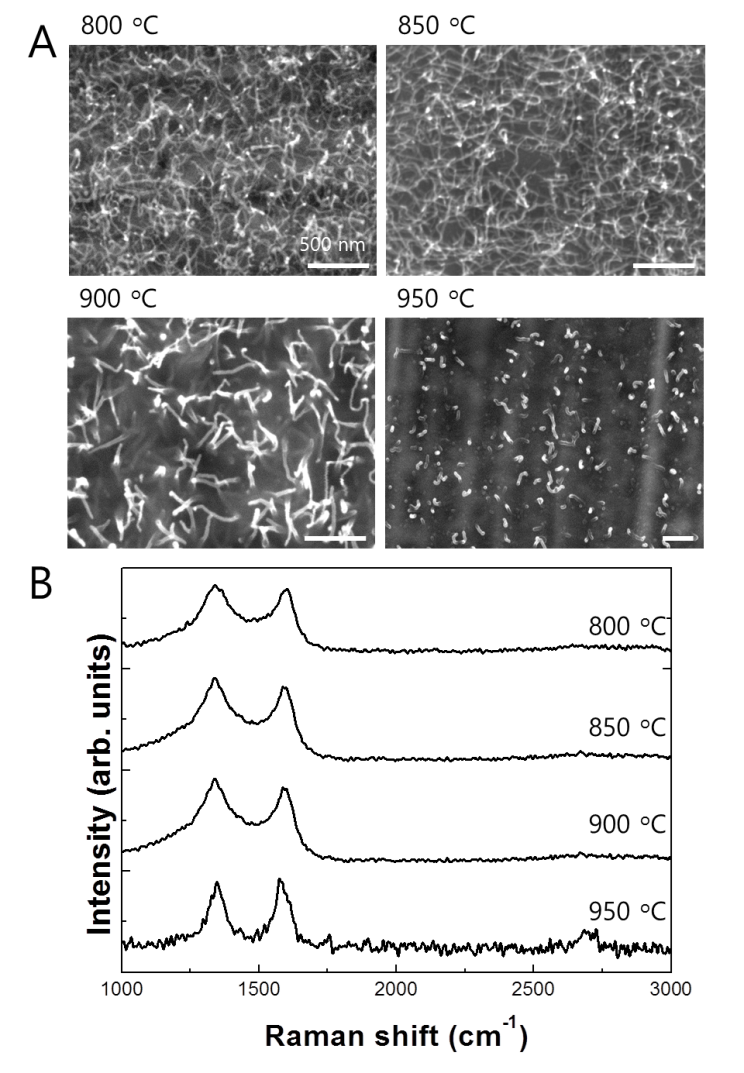


Figure S4. 3D CNTs/G morphology and Raman spectra changes with increasing growth temperatures. (A) SEM images of 3D CNTs/G networks grown at temperatures between 800 ^o^C and 950 ^o^C. At growth temperatures of 800 ^o^C and 850 ^o^C, long CNTs are observed but it is difficult to control the length of CNTs due to a rapid growth rate. At growth temperatures of 900 ^o^C and 950 ^o^C, the diameter of CNTs significantly increased while the length decreased. (B) Raman spectra of the 3D CNTs/G networks obtained from 800 ^o^C to 950 ^o^C. There is no remarkable difference on their structural quality despite we choose higher growth temperatures. D/G ratios in Raman from 800 ^o^C to 900 ^o^C remained almost the same. The growth temperature of 950 ^o^C is not suitable for the 3D CNTs/G due to their short and sparse structure.

Figure S5. Optical property and device performance of ultrathin graphite and the 3D CNTs/G networks. (A) Transmittance data of ultrathin graphite and the 3D CNTs/G networks by UV-Vis spectroscopy. Conventional graphene grown at high temperature with methane shows transmittance of ~97 % at 550 nm, while ultrathin graphite and the 3D CNTs/G grown at low temperature with acetylene show transmittance of ~88 % and ~82 %, respectively. (B) The current density-voltage curves of the perovskite structures with the only-ultrathin graphite and the 3D CNTs/G. The conversion efficiencies of ultrathin graphite and the 3D CNTs/G based devices are 5.54 % and 7.88 %, respectively. This relative low efficiency can be explained by several aspects including reduced transmittance by the 3D CNTs/G and the relatively thick active layer to prevent an electrical short by covering the 3D CNTs/G. For consideration of low transmittance, an inverted device structure avoiding light absorption through the 3D CNTs/G or an insertion in HTL instead of ETL will be beneficial to improve their performance. In addition, the thickness of the 3D CNTs/G should be optimized with electron- and hole-diffusion lengths for the effective carrier collection. Although there are several improvement points, the 3D CNTs/G obviously provided high surface area and fast lifetime of carriers and its structure showed 42% enhancement compared to the ultrathin graphite based structure. The inset shows the cross-sectional SEM image of the 3D CNTs/G based structure (It is difficult to distinguish the CNTs/G after the deposition of the active layer).
